# The Effect of Childhood Trauma on the Alleviation of Transdiagnostic Depressive Symptoms and the Mediating Role of Resilience in Outpatient Adolescents

**DOI:** 10.1007/s40653-025-00728-8

**Published:** 2025-09-16

**Authors:** Katriina M. Sarnola, Siiri-Liisi Kraav, Virve Kekkonen, Petri Kivimäki, Sebastian Therman, Tommi Tolmunen

**Affiliations:** 1 Department of Psychiatry, Institute of Clinical Medicine, University of Eastern Finland, P.O. Box 1627, 70211 Kuopio, Finland; 2 Department of Psychiatry, Kuopio University Hospital, P.O. Box 100, 70211 Kuopio, Finland; 3 Department of Social Sciences, University of Eastern Finland, 1627, 70211 Kuopio, Finland; 4 Institute of Behavioural Sciences (Psychology), University of Helsinki, P.O. Box 9, 00014 Helsinki, Finland

**Keywords:** Adolescent, Childhood trauma, Depression, Resilience, Transdiagnostic

## Abstract

Childhood traumatic experiences have long-term negative consequences for individual mental and physical well-being. Specifically, depression has proven to be a common consequence of previous trauma in adolescents. Furthermore, the course of depressive symptoms is worse in individuals with a traumatic history. Resilience may mitigate the impact of different types of trauma on depressive symptoms, but there is less information about these associations longitudinally. We therefore examined the effect of childhood trauma, including physical and emotional neglect and abuse, on the change in depressive symptoms over a six-month follow-up in outpatient adolescents, as well as the role of resilience in this association. We used a transdiagnostic approach by examining the depressive symptoms, resilience, and childhood traumatization, regardless of the psychiatric diagnosis, in a naturalistic study setting. Resilience measured with the Brief Resilience Scale (BRS), childhood adversities and trauma experiences, including emotional neglect, emotional abuse, physical neglect, physical abuse, and sexual abuse, measured with the Trauma and Distress Scale (TADS), and depressive symptoms measured with the Beck Depression Inventory (BDI-IA) were self-evaluated at baseline and on six-month follow-up by 297 adolescent participants in a natural study setting. Paired sample t-tests were conducted to measure the change in BDI scores during the follow-up. Mediation and moderation analysis with resilience was performed to investigate the effect of resilience on the association between trauma subtypes and the change in depressive symptoms during the follow-up. Childhood adversities and trauma experiences associated with resilience, the strongest associations being with emotional abuse, emotional neglect, and physical neglect, as well as with the alleviation of depressive symptoms during the follow-up, except for sexual abuse. The latter effect was mediated by resilience, even after adjustment for several possible confounding factors. Only sexual abuse did not associate with the change in depressive symptoms through resilience. No moderating effect of resilience was observed on the association between trauma or any trauma subtype and the alleviation of depressive symptoms. The broad assessment of childhood traumatic experiences, including emotional neglect, emotional abuse, physical neglect, and physical abuse, together with resilience in relation to depressive symptoms, regardless the psychiatric diagnosis, followed by interventions focused on resilience enhancement is suggested to alleviate depression in outpatient adolescents with such experiences of trauma.

**Supplementary Information:**

The online version contains supplementary material available at 10.1007/s40653-025-00728-8.

## Introduction

Childhood traumatic experiences have long-term negative consequences for psychological functioning, adaptation, and mental and physical well-being (Fairbank & Fairbank, 2009; Felliti et al., 1998). Prolonged stress responses to early traumatic experiences may cause changes in brain structure and development, predisposing individuals to developing mental health problems (Heim & Nemeroff, 2001; Shonkoff & Garner, 2012). Childhood trauma has also been proven to be linked to negative psychological functioning, such as harmful coping mechanisms, and impaired resilience, which can predispose an individual to mental health problems (Browne & Winkelman, 2007; Zhao et al., 2022).

Childhood traumatic experiences are transdiagnostic risk factors for different mental health problems in both adults and adolescents (Breslau et al., 2014; Infurna et al., 2016; Li et al., 2016; Palmier-Claus et al., 2016; Quide et al., 2020). For example, adolescents exposed to traumas are at greater risk of depression and anxiety (Lee et al., 2020), and cumulative trauma exposure heightens the risk of depression, anxiety, and their comorbidity in the young (Elmore et. al., 2020; Qu et al., 2022). Other possible consequences associated with early trauma may include adult-onset depression, the recurrence of depression, major depressive disorder, bipolar 1 and bipolar 2 disorders, anxiety disorders, and post-traumatic stress disorder (Breslau et al., 2014; Infurna et al., 2016; Li et al., 2016; Palmier-Claus et al., 2016; Quide et al., 2020). Childhood trauma is also linked to a worse clinical course of mental illness, including an earlier age of onset, episode persistence and recurrence, suicide attempts, suicide risk, and treatment resistance in adults (Danese & Widom, 2023; Douglas & Porter, 2012; Hovens et al., 2012; Tunnard et al., 2014). Among adolescents, individuals without traumatic experiences fare better when receiving treatment for depression (Waldron et al., 2019).

However, a common consequence of trauma for children and adolescents is depression (Vibhakar et al., 2019). Depression is a major global health concern among youth, with prevalence estimates of 21.3% for mild-to-severe depression, 18.9% for moderate-to-severe depression, and 3.7% for major depression (Lu et al., 2024). In addition, depressive symptoms are considered to be clinically crucial transdiagnostic symptoms in psychiatric disorders other than mood disorders (Nakajima et al., 2023). Depressive symptoms affect the quality of life and are associated with the risk of suicidal behavior and functional impairment (Garcia-Estela et al., 2024; Nakajima et al., 2023). Because depressive symptomatology can be present in all psychiatric disorders and worsen their clinical and functional outcomes, it is important to examine depressive symptoms using a transdiagnostic approach instead of only observing them categorically based on the specific psychiatric diagnosis (Garcia-Estela et al., 2024). The transdiagnostic approach considers that many psychiatric symptom dimensions are not restricted to a single diagnostic category but are shared, and that there is significant comorbidity between psychiatric disorders (Wise et al., 2022), especially among adolescents (Garber & Weersing, 2010).

To prevent the negative consequences of traumatic experiences for mental health and possibly prevent mental illness in the young, it is important to gain a better understanding of the underlying mechanisms linking traumatic experiences and depressive symptoms in adolescence. However, not everyone exposed to childhood trauma develops mental health problems such as depressive symptoms. This is partially explained by resilience, often defined as a defense and positive adaptation despite adversity (Masten, 2001). Resilience has been found to be a protective factor for adolescent mental health, for example, by mitigating the impact of traumatic experiences on the risk of depression (Bethell et al., 2014; Ding et al., 2017; Masten, 2001). According to adolescent resilience theory, the effect of resilience comes from its internal assets and/or external resources, and childhood traumatic events may hinder both, leading to reduced resilience (Fergus & Zimmermann, 2005; Ji, 2024).

Resilience and childhood traumatic experiences also appear to be interrelated, as resilience is negatively associated with childhood trauma and its subtypes, specifically with emotional abuse, emotional neglect, and sexual abuse (Kesebir et al., 2015; Park et al., 2023). Emotional abuse and emotional neglect display more robust relationships with resilience and are linked to lower levels of resilience in both psychiatric patients and control groups (Park et al., 2023).

There is evidence of resilience as a protective factor against psychopathology caused by earlier trauma (Campbell-Sills et al., 2006; Chang et al., 2021; Ding et al., 2017; Goldenson et al., 2021; Poole et al., 2017; Vieira et al., 2020; Wang et al., 2020; Wingo et al., 2010; Yu et al., 2021). For example, in a cross-sectional study by Vieira et al. (2020), a lack of resilience mediated the association of childhood trauma with mood disorders and depressive symptoms in a community sample of young adults, but also in those with major depressive disorder (MDD) and bipolar disorder (BD). In another striking example, individuals with significant childhood trauma combined with high resilience reported the lowest rates of psychiatric symptoms in a clinical sample of college students (Cambell-Skills et al., 2006). Similarly, Goldenson et al. (2021) found that resilience moderated the effect of trauma on depression and somatization in adolescents. One study detected no moderating effect of resilience on this association, possibly because of a lack of variance in resilience among the study participants (Freeny et al., 2021).

Resilience can be impaired by childhood adversity, leading to depression, or act as a buffering factor for mental health against adverse events (Elmore et al., 2020, 2023; Park et. al., 2023; Zhao et al., 2022). Resilience, or the lack thereof, may be one of the mechanisms explaining the association between childhood traumatic experiences and depressive symptoms. However, most earlier studies have been conducted cross-sectionally and have explored the association between childhood traumatic experiences, depression, and resilience either through the mediation or the moderation of resilience, or both (Campbell-Sills et al., 2006; Chang et al., 2021; Ding et al., 2017; Elmore et al., 2020; Poole et al., 2017; Vieira et al., 2020; Wang et al., 2020; Wingo et al., 2010; Yu et al., 2021). Moreover, there is a lack of information on the impact of childhood traumatization on the alleviation of depressive symptoms and the role of resilience in this association. There is also a need for more research on the different trauma subtypes and their specific impacts on mental health in young people.

### Current study

Given these previous findings and based on the transdiagnostic approach, a Link between childhood traumatic experiences, resilience, and depressive symptoms could also be found in heterogeneous psychiatric adolescent outpatients. However, to our best knowledge, we are the First to examine these associations longitudinally in a naturalistic study setting. If an association between childhood traumatic experiences, resilience, and depressive symptoms exists in this heterogeneous psychiatric patient group, it may be possible to reduce their depressive symptoms via resilience enhancement, despite the specified psychiatric diagnosis. Overall, our study compared the association of resilience with childhood trauma subtypes and the Change in depressive symptoms over six months of naturalistic clinical follow-up among heterogeneous outpatient adolescents. Our hypotheses were that 1) higher levels of childhood trauma would be negatively associated with resilience, 2) which in turn would be associated with a smaller change in depressive symptoms during the naturalistic six-month follow-up. We exploratively examined the different trauma subtypes separately in this association and conducted mediation and moderation analyses to assess whether there is any buffering effect of resilience against the negative impact of childhood trauma subtypes on depressive symptoms during the follow-up.

## Methods

### Participants and Procedures

This study was a secondary data analysis of the REAL-SMART project ("Recognition and early intervention for alcohol and substance abuse in adolescence and systemic alterations related to different psychiatric disease categories in adolescent outpatients"), the primary aim of which was to evaluate various psychiatric diagnostic profiles and predict the sequelae of the psychiatric state in adolescence with different methods, such as metabolomics.

In the present study, all 14–20-year-old patients (*n* = 2716) who were admitted to the adolescent psychiatric outpatient clinic of Kuopio University Hospital between June 2017 and June 2023 were approached. The only exclusion criterion was the inability to understand the Finnish-language study questionnaires. At baseline, consenting participants were interviewed and asked to complete self-rated questionnaires on a laptop computer. The interview included the Structured Clinical Interview for DSM disorders IV (SCID-IV; First et al., 2007), which was performed by a trained psychiatric nurse. The participants were interviewed again on six-month follow-up and completed an identical set of self-rated questionnaires on an Android tablet computer using OpenODK Collect (Hartung et al., 2010). Answering the study Questionnaires took approximately two hours per occasion. There was no compensation for participation in the study. All participants provided written informed consent, and the consent was also approved by the caregivers of participants who were under 15 years old at the study baseline, as mandated by national guidelines. The parents of minors aged 15 or older were informed of the study.

During the study, there were some periods during which no study nurses were available to perform the follow-up assessments, which naturally increased the dropout rate between the baseline and the follow-up. The COVID-19 pandemic also affected the dropout rate, because there were lockdown periods during the study. We were unable to reach 47 of 699 study participants for the follow-up. The mental health status and the treatment received by the study participants might have influenced the dropout rate, because there were participants who were no longer receiving psychiatric treatment from the outpatient clinic.

We restricted the analyses to examining data from those who had completed all measures at baseline and on six-month follow-up. Of the baseline cohort, 2019 refused to participate, leaving a total of 699 participants, of whom 297 had completed all the questionnaires at both time points (at baseline and on six-month follow-up) by June 2023.

Of the participants in the final dataset, 69 were male and 228 were female, and their mean age was 16.5 (SD 1.6, range 13–19) years. During the follow-up period, all participants received psychiatric treatment as needed in a naturalistic clinical setting. All participants received ongoing clinician-based case management and relevant social, psychological, and medical treatments as part of standard care. This may have involved contact with a psychiatrist, psychologist, therapist, or social support worker and hospitalization for patients whose needs exceeded the capacity of the outpatient clinical services.

The study conformed to the standards set by the 7th revision of the Declaration of Helsinki (World Medical Association, 2013). The study protocol was approved by the Kuopio University Hospital Research Ethics Committee.

In Finland, adolescent psychiatric treatment is free of charge. Adolescents may be referred to psychiatric examination and treatment from public health care, private health care, and by school doctors. Some adolescents may receive their whole treatment in the private sector, especially if their psychiatric problems are not very severe. There are no exact numbers of those treated in the private sector and public health care.

### Questionnaires

#### Depression

Depressive symptoms were assessed with the Beck Depression Inventory-IA (BDI-IA; Beck et al., 1979). The BDI is a 21-item measure of current depressive symptoms, which is widely used globally and is known to have strong psychometric properties (Beck et al., 1996; Steer et al., 1998; Stockings et al., 2015). Items are scored from 0–3 and are summed for a total score of 0–63. The Cronbach alpha measure of internal consistency for the BDI was .93 at baseline and .95 on follow-up. Stockings et al. (2015) demonstrated in their meta-analysis that the BDI, among other commonly used depression symptom rating scales, is a reliable measure of depressive symptoms among adolescents: the pooled estimate for internal reliability in their study was .86 (12 data points, *n* = 4152, 95% CI: [.81,.90]), classified as ‘good’. In reporting sum scores, missing values were replaced by the respondent’s mean for the other items at that time point.

#### Childhood Traumatic Experiences

The Trauma and Distress Scale (TADS) was used to assess five types of childhood adversities and traumas, namely Lifetime emotional abuse, emotional neglect, physical abuse, physical neglect, and sexual abuse, with five items for each subscale, as well as a total score employing all 43 items (Patterson et al., 2002; Salokangas et al., 2016). The TADS items are rated on a five-point rating scale, ranging from 0 = “never” to 4 = “almost always”. It has proven to be a valid, reliable, and clinically useful instrument for retrospectively assessing childhood traumatization (Kurkinen et al., 2023; Porthan et al., 2023; Salokangas et al., 2016; 2020). The Cronbach alpha for the full scale was .80 at baseline and .82 on follow-up. Salokangas et al. (2016) found in their general adult population sample that the internal consistency of the 25 items in the five domains (used to calculate the TADS total sum score) was .92.

#### Resilience

The Brief Resilience Scale (BRS; Smith et al., 2008) was used to assess self-reported trait resilience. The BRS has six items, including “I tend to bounce back quickly after hard times” and “I have a hard time making it through stressful events”. High BRS scores appear to be uniquely related to health, and especially to reducing anxiety, depression, negative affect, and physical symptoms (Smith et al., 2008). The BRS has earlier demonstrated good internal consistency, convergent validity, and discriminant validity (Smith et al., 2008), including in individuals with mental disorders (Broll et al., 2024; Sanchez et al., 2021). In this study, the Cronbach alpha was .88 at baseline and .88 on follow-up. Similarly, Broll et al. (2024) found in their clinical sample of psychiatric inpatient adults that the internal consistency of the BRS was good (α =.79, 95% CI [.77;.80]). In a study by Sanchez et al. (2021), the BRS alpha was .87 and .85 in their clinical samples of adults with serious mental illness.

### Statistical Analysis

#### Missingness Analysis

As there was major attrition between baseline and follow-up, we compared background variables (age, sex) and the baseline clinical variables of interest (depression, resilience, and trauma levels) between the follow-up participants (*n* = 319) and those lost to follow-up (*n* = 380) using with nonparametric permuted Brunner-Munzel test, a.k.a. the Generalized Wilcoxon Test, as implemented in the brunnermunzel package (v.2.0; Ara 2022) for R (v.4.4.2; R Core Team, 2022). The probability of superiority effect sizes and their confidence intervals were computed with the same package. Correlations between the clinical variables at baseline were compared with two-sided Fisher’s z tests as implemented in the cocor package (v. 1.1.−4; Diedenhofen & Musch, 2015) for R. Due to multiple testing, the *p*-value for statistical significance in the missingness analyses was set to .01.

#### Factor analysis

To obtain normalized and more accurate individual scores, we performed factor analysis on the responses of the 699 baseline participants to estimate factor scores for further analyses to improve the distributions and measurement precision over the sum scores. Single- and five-factor item a priori factor analyses of the 25 baseline TADS items forming the five subscales were estimated with Mplus v8.8 (Muthén & Muthén, 2017) using the WLSMV estimator, theta parameterization, 1000 random starts, and otherwise standard settings. WLSMV, the default estimator in Mplus for ordinal data, is appropriate when analyzing rating scales with a model including response thresholds and outperforms maximum likelihood when indicators are ordinal and the latent distribution is approximately normal (Flora & Curran, 2004). Model fit was assessed with the criteria of Hu and Bentler (1999), which are appropriate when factor loadings are around.7 (McNeish et al., 2018): root-mean-square error of approximation (RMSEA) <.06, comparative fit index (CFI) >.95, and standardized root-mean-square residual (SRMR) <.08. Factor scores were obtained with the maximum a posteriori method. Missingness was negligible for the cases with any data, with a covariance coverage of 98.3% or higher for all item pairs, and the estimation method used all pairwise information. Single-dimensional BDI and BRS factor scores were estimated in the same way. For comparability, follow-up factor scores were generated using the baseline model parameters, except for factor means and variances, which were freed.

The correlation analysis and the mediation and moderation analyses were performed using factor scores of resilience (BRS) and trauma experiences (TADS) instead of sum scores for improved measurement properties. Descriptive statistics for all variables and Pearson correlations between variables are presented in Tables 1 and 2. To test the change in BDI scores, we used the paired-sample *t*-test. Simple mediation and moderation analyses were performed with the PROCESS macro v.4.1 for SPSS (Hayes, 2018). The standard number of bootstrap samples (5000) was used. If the upper and lower bounds of 95% confidence intervals did not contain zero, the indirect effect was considered significant. In all mediation and moderation analyses, age and gender were used as covariates. Data were analyzed using SPSS 27.0.1, and *p*-values under.05 were considered statistically significant.

**Table 1:** Descriptive statistics for the continuous variables (*n* = 297)

Variable	Min	Max	Mean	SD
Age, years (baseline)	13	19	16.52	1.64
Beck Depression Inventory sum score (BDI)				
Baseline (BL)	0	52	22.05	12.39
Follow-up (F/U)	0	52	18.59	13.25
Change (Δ)	−44	40	−3.46	10.77
Brief Resilience Scale baseline mean score (BRS)	1	5	2.79	0.82
TADS baseline sum scores				
Total	2	116	38.57	20.35
EmoAb	0	19	4.65	3.97
EmoNg	0	20	6.98	4.19
PhyNg	0	15	3.66	2.86
PhyAb	0	15	1.67	2.58
SexAb	0	16	1.37	2.81
TADS baseline factor scores				
Single-factor model (1f)	−2.42	2.48	−0.12	0.95
EmoAb	−2.14	2.87	−0.12	0.93
EmoNg	−2.18	3.02	−0.08	0.97
PhyNg	−2.25	3.41	−0.11	0.96
PhyAb	−1.80	3.38	−0.12	0.95
SexAb	−1.42	3.20	−0.12	0.93
BRS baseline factor score	−2.59	2.92	0.03	1.02

*BDI* Beck Depression Inventory-IA, *BRS* Brief Resilience Scale, *TADS* Trauma and Distress Scale, *EmoAb* Emotional Abuse, *EmoNg* Emotional Neglect, *PhyNg* Physical Neglect, *PhyAb* Physical Abuse, *SexAb* Sexual Abuse

**Table 2:** Pearson correlation coefficients between the sum and mean variables (BDI BL, BDI F/U, BDI Δ, TADS total BL), BRS single-factor score and TADS single-factor and subscale factor scores used in the mediation and moderation analyses

	BDI BL	BDI F/U		BDI Δ		BRS		TADS total		TADS 1f		Emo Ng		Emo Ab		Phy Ng		Phy Ab		Sex Ab	
Age	.07	-.07		-.16***		-.12*		.14*		.16***		.13*		.16**		.15**		.15**		.16**	
BDI BL		.65***		-.35***		-.54***		.49***		.42***		.35***		.44***		.37***		.31***		.31***	
BDI F/U				.48***		-.34***		.34***		.29***		.23***		.30***		.23***		.17**		.25***	
BDI Δ						.20***		-.14*		-.13*		-.12*		-.14*		-.15*		-.15*		-.05	
BRS								-.32***		-.27***		-.25***		-.32***		-.24***		-.18**		-.12*	
TADS total										.91***		.83***		.92***		.90***		.81***		.59***	
TADS 1f												.91***		.92***		.96***		.79***		.66***	
EmoNg														.87***		.96***		.59***		.33***	
EmoAb																.90***		.76***		.54***	
PhyNg																		.79***		.48***	
PhyAb																				.64***	

*BDI* Beck Depression Inventory-IA, *BRS* Brief Resilience Scale, *TADS* Trauma and Distress Scale, *EmoNg* Emotional Neglect, *EmoAb* Emotional Abuse, *PhyNg* Physical Neglect, *PhyAb* Physical Abuse, *SexAb* Sexual Abuse, *BL* Baseline, *F/U* Follow-up, Δ = Change, 1f = Single-Factor Score

*p <.05; **p <.01; ***p <.001

## Results

### Missingness Analysis

The follow-up participants and those lost to follow-up had very similar age, BDI, BRS, and TADS distributions at baseline (Supplementary Table 6). The largest group difference in baseline characteristics was in the gender distribution (77% vs. 70%), but this did not reach statistical significance. The baseline correlations between clinical variables (Supplementary Table 7) were also statistically nonsignificant.

### Factor Analysis

The estimated single-dimensional BDI (baseline and follow-up) and BRS factor scores provided a good fit to the data based on the CFI and SRMR indices, as did the a priori five-dimensional model of the TADS, although only the latter had a good RMSEA. The single-dimensional TADS had a poor fit, although the factor model was an improvement on the sum score model. The fit of each of the factor models is provided in Supplementary Table 1.

### BDI Sum Score Change

Compared to the levels at baseline, BDI scores were lower on 6-month follow-up. BDI mean sum scores for the whole sample (*n* = 297) were 22.05 (SD 12.39) at baseline and 18.59 (SD 13.25) on 6-month follow-up, and the change in BDI mean sum scores over time was −3.47 (SD 10.77). The *t*-test value for the paired sample *t*-test was 5.55 (*p* <.001) and the effect size of the change (Cohen’s *d*) was 0.32.

### Correlations Between Variables

The resilience factor score measured at baseline displayed a moderate negative correlation with depressive symptoms at baseline and on 6-month follow-up. The correlation between the baseline resilience factor scores and the change in depressive symptoms during the follow-up was positive and weak but statistically significant. The baseline resilience factor score correlated negatively with traumatic experiences measured with the TADS by showing the strongest correlation with the TADS total factor score, emotional abuse factor score, emotional neglect factor score, and physical neglect factor score. The correlations between the TADS total factor score, emotional abuse factor score, emotional neglect factor score, and physical neglect factor score with the baseline resilience factor score were negative and weak. Physical abuse and sexual abuse factor scores also displayed weak negative correlations with baseline resilience factor scores. The total trauma factor score correlated negatively and weakly with the change in depressive symptoms, and the correlations were quite similar between the different trauma subtypes and the change in depressive symptoms. However, there was no correlation between sexual abuse factor scores and the change in depressive symptoms. Age was weakly associated with the change in depression and with all the different trauma factor scores (Table 2).

### Mediating and Moderating Effect of Resilience

**Fig. 1:**
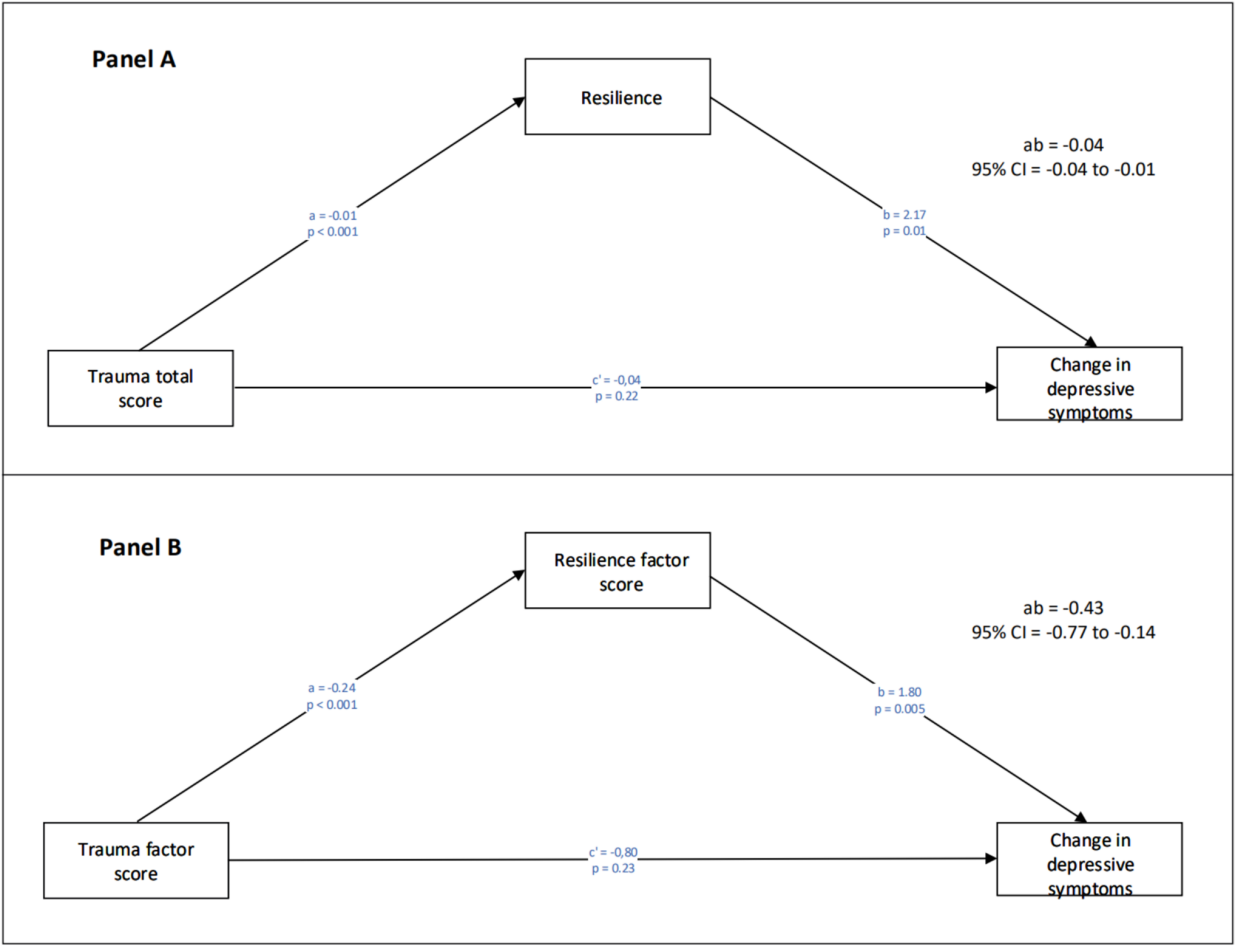
Panel A: The effect of trauma (TADS sum) on the change in depressive symptoms (BDI sum change) through resilience (BRS sum). Panel B: The effect of trauma (TADS total factor score) on the change in depressive symptoms (BDI sum score change) through resilience (BRS factor score). The results are adjusted for two possible confounders: age at baseline (U_1_) and gender (U_2_). Information on the confounding effect is presented in Table 3

In the simple mediation analysis, traumatic experiences, both as the total score and overall factor score, indirectly affected the level of change in depressive symptoms through resilience (Table 3, Fig. 1, panels A and B). This was also the case when the trauma subtypes were considered (Table 4). Only sexual abuse factor scores did not associate with the change in depressive symptoms during the follow-up through resilience (Table 4). Traumatic experiences were negatively associated with resilience (*a* = −0.01), which in turn positively affected the change in depression between baseline and follow-up (*b* = 2.17). The 95% bootstrap confidence interval for the indirect effect (*ab* = −0.03) was entirely below zero (−0.04; −0.01). There was no evidence of trauma factor scores influencing the change in depression independently of resilience (c'= −0.04, *p* =.22). We conducted the same type of moderation analysis on the interrelationships between traumatic experiences, resilience, and the change in depressive symptoms. These analyses indicated no moderating effect of resilience on the association between childhood adversity and trauma and the change in depressive symptoms (Supplementary Table 5). All the mediation and moderation analyses were adjusted for age and gender.

**Table 3:** Regression coefficients, standard errors (SE), and model summary information for simple mediation models assessing a) the effect that the baseline TADS total score (X) exerts on the change in the BDI sum score (Y) through the BRS sum score (M) and b) the effect that the baseline TADS factor score (X) exerts on the change in the BDI sum score (Y) through the BRS factor score (M). The results are adjusted for two possible confounders: age at baseline (U_1_) and gender (U_2_)

a. Sum scores model								
Antecedent	Consequent							
	Mediator (BRS)				Dependent (BDI change)			
		Coeff	*SE*	*p*		Coeff	*SE*	*p*
X (TADS)	*a*	−0.01	0.00	<.001	*c’*	−0.04	0.03	.22
M (BRS)		–	–	–	*b*	2.17	0.82	.01
U_1_ Age	*a*_*2*_	−0.06	0.03	.03	*c´*_*2*_	−0.83	0.38	.03
U_2_ Gender	*a*_*3*_	−0.50	0.10	<.001	*c’*_*3*_	1.19	1.51	.43
Constant	*i*_*M*_	4.60	0.46	<.001	*i*_*y*_	3.03	6.30	.63
		R^2^ =.18				R^2^ =.06		
		*F*(21.21) = 293, *p* <.001				*F*(4.94) = 292, *p* <.001		
		ab = −0.03; Boot SE = 0.01; 95% CI (boot) = −0.04 to −0.01						
b. Factor scores model								
Antecedent	Consequent							
	Mediator (BRS)				Dependent (BDI change)			
		Coeff	*SE*	*p*		Coeff	*SE*	*p*
X (TADS)	*a*	−0.24	0.06	<.001	*c’*	−0.80	0.67	.23
M (BRS)		–	–	–	*b*	1.80	0.64	.01
U_1_ Age	*a*_*2*_	−0.07	0.03	.03	*c´*_*2*_	−0.83	0.38	.03
U_2_ Gender	*a*_*3*_	−0.62	0.13	<.001	*c’*_*3*_	1.35	1.51	.37
Constant	*i*_*M*_	1.68	0.59	.01	*i*_*y*_	9.15	6.59	.17
		R^2^ =.14				R^2^ =.06		
		*F*(16.17) = 295, *p* <.001				*F*(5.02) = 294, *p* <.001		
		ab = −0.43; Boot SE = 0.16; 95% CI (boot) = −0.77 to −0.14

**Table 4:** Regression coefficients, standard errors (SE), and summary information for the simple mediation model assessing the effect that baseline TADS subscale factor scores (X) exert on the change in the BDI sum score (Y) through the BRS baseline factor score (M)

a. Emotional Abuse												
Antecedent	Consequent											
	Mediator (BRS)							Dependent (BDI change)				
		Coeff		*SE*		*p*			Coeff	*SE*	*p*	
X (EmoAb)	*a*	−0.31		0.06		<.001		*c’*	−0.82	0.69	.24	
M (BRS)		–		–		–		*b*	1.76	0.65	.01	
U_1_ Age	*a*_*2*_	−0.07		0.03		.05		*c´*_*2*_	−0.83	0.38	.03	
U_2_ Gender	*a*_*3*_	−0.60		0.13		<.001		*c’*_*3*_	1.33	1.51	.38	
Constant	*i*_*M*_	1.55		0.58		.01		*i*_*y*_	9.23	6.58	.16	
		R^2^ =.17							R^2^ =.06			
		*F*(295) = 19.97, *p* <.001							*F*(294) = 5.01, *p* <.001			
ab = −0.54; Boot SE = 0.18; 95% CI (boot) = −0.92 to −0.18												
b. Emotional Neglect												
Antecedent	Consequent											
	Mediator (BRS)							Dependent (BDI change)				
		Coeff		*SE*		*p*			Coeff	*SE*	*p*	
X (EmoNg)	*a*	−0.24		0.06		<.001		*c’*	−0.67	0.65	.30	
M (BRS)		–		–		–		*b*	1.82	0.64	.005	
U_1_ Age	*a*_*2*_	−0.08		0.03		.02		*c´*_*2*_	−0.85	0.38	.02	
U_2_ Gender	*a*_*3*_	−0.65		0.13		<.001		*c’*_*3*_	1.24	1.51	.41	
Constant	*i*_*M*_	1.80		0.58		.002		*i*_*y*_	9.71	6.55	.14	
		R^2^ =.14							R^2^ =.06			
		*F*(295) = 16.64, *p* <.001							*F*(294) = 4.92, *p* <.001			
ab = −0.44; Boot SE = 0.17; 95% CI (boot) = −0.80 to −0.14												
c. Physical Abuse												
Antecedent	Consequent											
	Mediator (BRS)						Dependent (BDI change)					
		Coeff	*SE*		*p*				Coeff	*SE*		*p*
X (PhyAb)	*a*	−0.18	0.06		.003		*c’*		−1.02	0.66		.12
M (BRS)		–	–		–		*b*		1.81	0.63		.004
U_1_ Age	*a*_*2*_	−0.08	0.03		.02		*c´*_*2*_		−0.82	0.38		.03
U_2_ Gender	*a*_*3*_	−0.68	0.13		<.001		*c’*_*3*_		1.17	1.51		.44
Constant	*i*_*M*_	1.87	0.59		.002		*i*_*y*_		9.10	6.55		.17
		R^2^ =.12							R^2^ =.07			
		*F*(295) = 13.35, *p* <.001							*F*(294) = 5.27, *p* <.001			
ab = −0.32; Boot SE = 0.14; 95% CI (boot) = −0.62 to −0.08												
d. Physical Neglect												
Antecedent	Consequent											
	Mediator (BRS)						Dependent (BDI change)					
		Coeff	*SE*		*p*				Coeff	*SE*		*p*
X (PhyNg)	*a*	−0.23	0.06		<.001		*c’*		−0.97	0.66		.14
M (BRS)		–	–		–		*b*		1.77	0.64		.006
U_1_ Age	*a*_*2*_	−0.07	0.03		.03		*c´*_*2*_		−0.83	0.38		.03
U_2_ Gender	*a*_*3*_	−0.62	0.13		<.001		*c’*_*3*_		1.20	1.51		.43
Constant	*i*_*M*_	1.77	0.59		.003		*i*_*y*_		9.25	6.55		.16
		R^2^ =.14							R^2^ =.07			
		*F*(295) = 16.04, *p* <.001							*F*(294) = 5.21, *p* =.001			
ab = −0.41; Boot SE = 0.16; 95% CI (boot) = −0.75 to −0.13												
e. Sexual Abuse												
Antecedent	Consequent											
	Mediator (BRS)						Dependent (BDI change)					
		Coeff	*SE*		*p*				Coeff	*SE*		*p*
X (SexAb)	*a*	−0.04	0.06		.51		*c’*		−0.22	0.68		.75
M (BRS)		–	–		–		*b*		1.97	0.63		.002
U_1_ Age	*a*_*2*_	−0.09	0.04		.009		*c´*_*2*_		−0.87	0.39		.02
U_2_ Gender	*a*_*3*_	−0.66	0.14		<.001		*c’*_*3*_		1.39	1.55		.37
Constant	*i*_*M*_	2.06	0.61		<.001		*i*_*y*_		9.88	6.68		.14
		R^2^ =.09							R^2^ =.06			
		*F*(295) = 10.29, *p* <.001							*F*(294) = 4.66, *p* =.001			
ab = −0.08; Boot SE = 0.13; 95% CI (boot) = −0.35 to 0.19												

The results are adjusted for two possible confounders: age at baseline (U_1_) and gender (U_2_)

## Discussion

### Main Findings

As far as we are aware, this is the first study to investigate the longitudinal associations between different trauma subtypes, resilience, and depressive symptoms in a naturalistic clinical setting among adolescents. In our analysis, resilience correlated negatively with all the studied traumatic experiences, showing the strongest correlation with the TADS total score and the trauma subtypes emotional abuse, emotional neglect, and physical neglect. The higher the trauma scores were, the less resilience the study participants showed. Physical abuse and sexual abuse displayed weak negative correlations with resilience. Factor scores for all trauma subtypes excluding sexual abuse correlated quite equally but weakly with the change in depressive symptoms. In the mediation analysis, all the trauma subtypes, excluding sexual abuse, indirectly affected the level of change in depressive symptoms through resilience after adjusting the model for age and gender. However, resilience did not moderate the effect of trauma or different trauma subtypes on the change in depressive symptoms in this adolescent outpatient sample.

### Comparison with Earlier Literature

In our study, different types of childhood traumatic experiences were associated with a smaller decrease in depressive symptoms during the naturalistic clinical follow-up. The higher the trauma score, the smaller was the change in depressive symptoms, and the trauma history thus hampered the recovery of patients from depressive symptoms. Except for sexual abuse, this association did not differ based on the trauma subtype, but emotional abuse, emotional neglect, physical abuse, and physical neglect were equally negatively associated with the change in depressive symptoms. Earlier studies have also demonstrated that a history of trauma affects the treatment response by slowing the recovery compared to adolescents without a trauma history (Waldron et al., 2019). However, in previous studies concerning specific treatment responses, the trauma type has significantly impacted the treatment response among depressed adolescents (Lewis et al., 2010; Shamseddeen et al., 2011).

Childhood adverse experiences and trauma were associated with lower levels of resilience among the outpatient adolescents. The higher the trauma score, the lower was the level of resilience, and the overall association between traumatic experiences and resilience was negative, in agreement with earlier studies (Elmore & Crouch, 2023; Goldenson et al., 2021; Kesebir et al., 2015; Kim et al., 2021; Morgan et al., 2021; Park et al., 2023; Vieira et. al., 2020). We found resilience to correlate with all the trauma subtypes, showing the strongest correlations with emotional abuse and emotional neglect, which is in line with previous studies in adults (Citak & Erten, 2021; Kesebir et al., 2015; Lee et. al., 2018; Park et al., 2023). We also found negative correlations between resilience and physical abuse, physical neglect, and sexual abuse. In contrast, the clinical study of Kesebir et al. (2015) revealed no association between resilience and either physical neglect or physical abuse in their clinical adult sample, which may be due the differences in the effects of physical neglect and abuse on adolescents compared to adults (Cohen & Thakur, 2021). Moreover, Park et al. (2023) found that sexual abuse was not associated with resilience in any clinical or non-clinical study participants, regardless of gender, and overall, the different diagnostic groups affected the relationship between childhood trauma and resilience (Park et al., 2023). However, in vulnerable youth, all these harmful experiences apart from the trauma subtype (i.e., abuse vs. neglect, emotional, sexual vs. physical) may have an influence on their resilience tendencies due the developmental stage (Beutel et al., 2017). Childhood trauma affects the ability to cope with emotions and may lead to negative psychological functioning and hamper the development of healthy coping mechanisms (Beutel et al., 2017; Citak & Erten, 2021; Kesebir et al., 2015; Park et al., 2023). Specifically, the ability to cope and regulate emotions in stressful circumstances may play a crucial role in the development of resilience (Compas et al., 2017).

Despite their history of childhood traumatic experiences, the adolescents of our study were still resilient enough to recover from their depressive symptoms. They showed an alleviation of depressive symptoms, and this association was mediated by resilience. Only in the case of sexual abuse did resilience not mediate the effect on the alleviation of depressive symptoms, and there was no significant correlation between sexual abuse and the change in depressive symptoms. This might be due to a lower number of participants who had experienced sexual abuse or underreporting of the experiences. It is also possible that sexual abuse affects depressive symptoms and their change through other pathways than resilience, or that sexual abuse in this particular patient group did not affect recovery from depressive symptoms. Sexual abuse during childhood and adolescence may have detrimental effects on an individual’s mental health by increasing the symptoms of depression, anxiety, suicidal ideation, suicide attempts, and substance abuse (Fergusson et al., 2013). The patients with experiences of childhood sexual abuse might have experienced a more severe course of depression, which in turn might have influenced their response to treatment for depression (Dongdong et al., 2020; Nanni et al., 2012).

In earlier studies, the correlation between sexual abuse and resilience has varied with respect to gender, the study population (general vs. clinical/high risk population), the trauma intervention method used (self-report vs. interview), and the clinical psychiatric diagnosis (Kesebir et al., 2015; Park et al., 2023; Sanjeevi et al., 2018). It has also been argued that the more salient forms of childhood adversity, such as emotional abuse and emotional neglect, are more closely related to internalizing depressive symptoms than physical abuse or sexual abuse (Gibb et al., 2007; Infurna et al., 2016; Mandelli et al., 2015), and that these maltreatment types in particular significantly affect resilience skills (Park et al., 2023).

The present longitudinal study suggested a mediating role of resilience in the association between childhood trauma and depressive symptoms (Chen et al., 2022). As in previous studies, childhood adversity led to higher depressive symptoms through a lower level of resilience, although most previous studies have been conducted cross-sectionally (Chang et al., 2021; Ding et al., 2017; Freeny et al., 2021; Han et al., 2018; Lee et al., 2018; Liu et al., 2019; Metel et al., 2019; Vieira et al., 2020). Overall, resilience can be impaired by childhood adversity and trauma, leading to depression (Zhao et al., 2022). One explanation for this might be that childhood trauma adversely affects resilience and attachment, possibly by hindering the trust and predictability needed for their development (Citak & Erten, 2021). Bowlby’s attachment theory states that the early relationship with the primary caregiver plays a crucial role in the development of mental representations of the self, the other, and the relationship between the two (Bowlby, 1982; Citak & Erten, 2021). Individuals growing up in environments with abusive and/or neglective caregivers may be at risk of developing a more negative self-model, maladaptive coping mechanisms, and problems in later social adjustment, all of which are closely related to resilience tendencies (Bowlby, 1982; Citak & Erten, 2021; Park et al., 2023). Similarly, adolescent resilience theory states that the effects of resilience come from internal assets, such as competence, coping skills, and self-efficacy, or external resources, such as parental support, adult mentoring, or community organizations that promote positive youth development (Fergus & Zimmermann, 2005; Ji, 2024). Childhood traumatic experiences and adverse events negatively affect both resilience resources, which may lead to reduced resilience (Ji, 2024).

Some previous studies have also found that resilience buffers against the effect of childhood adversity on depression, acting as a moderator (Chang et al., 2021; Ding et al., 2017; Ji, 2024; Poole et al., 2017; Wang et al., 2020; Wingo et al., 2010; Yu et al., 2021). However, we only found a mediating effect of resilience and, in our study, resilience did not moderate the effect of childhood adversity and trauma on the change in depressive symptoms. Similarly, one earlier study did not detect a moderating effect of resilience in this association (Freeny et al., 2021). In our study, the participants already suffered from mental health conditions, which may have affected the results by lowering their resilience levels so that resilience was not sufficient to buffer the effect of childhood traumatic experiences on the alleviation of depressive symptoms.

### Strengths and Limitations

Compared to the earlier literature, our study was the first to examine childhood trauma subtypes separately in the context of resilience and the alleviation of depressive symptoms in adolescent outpatients by using a transdiagnostic approach. Our study design was a naturalistic clinical follow-up, and we did not split the data based on either psychiatric diagnoses or more specific psychiatric symptoms in order to keep all the studied variables continuous and the setting naturalistic and heterogeneous. The transdiagnostic approach allowed us to examine depressive symptoms, childhood traumatization, and resilience dimensionally, and these commonalities across psychiatric disorders are possibly important for their assessment and treatment.

Moreover, we conducted this study longitudinally, which is a key strength compared to earlier studies on the topic. The longitudinal study design allowed us to evaluate the change in depressive symptoms and draw possible causal conclusions. Moreover, besides the total trauma score, we took into account all of the trauma subtypes separately to explore their impact on the alleviation of depressive symptoms and the role of resilience in this association.

The study participants represented an ethnically homogeneous Caucasian origin, allowing us to interpret the findings only in this population. Since all the patients were outpatients in a university hospital, to which they were usually referred by primary practitioners, potential selection bias related to recruitment may limit the generalization of our findings. Thus, it is still unclear how well these results can be generalized to other populations, such as healthy adolescents, adults, and various population subgroups.

As a result of the COVID-19 pandemic, the study was interrupted several times. The follow-up time of the study was relatively short, so the effect of childhood adversities and trauma on depressive symptoms needs to be further investigated in longitudinal research settings with longer follow-up times to determine the long-term impact of traumatic experiences and resilience on depressive symptoms.

Self-report and prospective measuring of childhood traumatic experiences, resilience, and depressive symptoms while the participants are suffering from mental health conditions may temper the reliability of the study results. With regards to the TADS scale, it is important to note that it assesses adverse childhood events and traumatization during childhood and adolescence, before the end of mandatory schooling. The participants in our study might also have experienced traumatization during their participation in the study, which might affect the interpretation of the longitudinality of our study findings.

Moreover, the unequal gender distribution of the study sample, significant dropout rate, and relatively low overall response rates may cause potential selection bias and should be considered in interpreting the present findings. Due to the naturalistic clinical study design, we did not consider several detailed clinical characteristics or psychosocial determinants that might potentially influence the change in depressive symptoms, such as the specific psychiatric diagnosis, medical treatment and its duration, family history, or social support.

## Conclusions

Our study demonstrated that childhood adversities and trauma, irrespective of the trauma subtype, hamper recovery from depressive symptoms, despite the specified psychiatric diagnosis. Except for sexual abuse, the association between all other forms of traumatic experiences, including emotional abuse, emotional neglect, physical abuse, and physical neglect, and the alleviation of depressive symptoms was mediated by resilience, even after adjusting for age and gender. In contrast, there were no moderating effects of resilience on the association between childhood traumatic experiences and the change in depressive symptoms.

The resilience of heterogeneous outpatient adolescents with a history of interpersonal trauma has a crucial role in their recovery from depressive symptoms. Resilience has proven to be useful for the treatment of mental health problems, and there are efficient interventions to promote resilience in children and adolescents (Broll et al., 2024; Dray, 2021). In particular, cognitive behavioral therapy (CBT)-based approaches are suggested to be promising resilience-focused interventions to reduce depressive symptoms in children and adolescents (Dray et al., 2017). In conclusion, improving the resilience of adolescents with childhood traumatic experiences using diverse strategies may be one way to not only alleviate but possibly prevent the detrimental effects of trauma on adolescents’ mental health.

## Supplementary Information

Below is the link to the electronic supplementary material.

Supplementary file1 (DOCX 36 KB)

Supplementary file2 (DOCX 22 KB)

## Data Availability

The data are available from the corresponding author upon request.
