# Supplementary material for: The Effect of Childhood Trauma on the Alleviation of Transdiagnostic Depressive Symptoms and the Mediating Role of Resilience in Outpatient Adolescents
Source: J Child Adolesc Trauma. 2025 Sep 16;18(4):1005–19. doi: 10.1007/s40653-025-00728-8 (PMC12831748; doi:10.1007/s40653-025-00728-8)
Supplement: Supplementary file 1 — Supplementary file1 (DOCX 36 KB) [file 40653_2025_728_MOESM1_ESM.docx]

Supplementary material

**Suppl. Table 1.** Model fit of factor models.

|  | Respondents with any data | Minimum item pair covariance coverage | Comparative Fit Index (CFI) | Root Mean Square Error of Approximation (RMSEA) | Standardized Root Mean Square Residual (SRMR) |
| --- | --- | --- | --- | --- | --- |
| BDI baseline | 697 | 89.2%^a^ | .953 | .085 | .053 |
| BDI follow-up | 305 | 95.4%^b^ | .971 | .072 | .069 |
| BRS baseline | 686 | 98.8% | .986 | .118 | .018 |
| TADS 1D baseline | 692 | 98.3% | .845 | .131 | .138 |
| TADS 5D baseline | 692 | 98.3% | .972 | .057 | .055 |

^a^ When involving item 19 (“Weight loss”), 98.1% otherwise.

^b^ When involving item 19 (“Weight loss”), 98.7% otherwise.

**Suppl. Table 2.** Standardized BDI factor model.

| Item | Loading | Thresholds | | |
| --- | --- | --- | --- | --- |
|  |  | 1 | 2 | 3 |
| 1 | .80 | -0.52 | 0.62 | 1.52 |
| 2 | .75 | -0.83 | 0.23 | 1.10 |
| 3 | .84 | -0.73 | 0.47 | 1.14 |
| 4 | .84 | -0.56 | 0.44 | 1.45 |
| 5 | .88 | -0.46 | 0.29 | 1.00 |
| 6 | .65 | 0.07 | 0.75 | 1.29 |
| 7 | .83 | -0.68 | 0.34 | 0.97 |
| 8 | .81 | -0.89 | -0.20 | 0.32 |
| 9 | .69 | -0.69 | 1.11 | 2.08 |
| 10 | .71 | -0.48 | 0.26 | 0.70 |
| 11 | .66 | -0.91 | 0.33 | 1.33 |
| 12 | .75 | -0.38 | 0.62 | 1.78 |
| 13 | .76 | -0.68 | 0.04 | 1.60 |
| 14 | .72 | -0.12 | 0.39 | 0.65 |
| 15 | .72 | -0.91 | -0.03 | 1.48 |
| 16 | .57 | -0.68 | 0.79 | 1.33 |
| 17 | .78 | -0.78 | 0.08 | 1.25 |
| 18 | .58 | -0.05 | 0.75 | 1.56 |
| 19 | .18 | 0.91 | 1.42 | 1.88 |
| 20 | .41 | 0.27 | 1.48 | 1.94 |
| 21 | .58 | 0.20 | 0.93 | 1.62 |

**Suppl. Table 3.** Standardized BRS factor model.

| Item | Loading | Thresholds | | | |
| --- | --- | --- | --- | --- | --- |
|  |  | 1 | 2 | 3 | 4 |
| 1 | .82 | -1.66 | -0.65 | 0.49 | 1.58 |
| 2 | -.72 | -1.71 | -0.86 | -0.07 | 1.15 |
| 3 | .77 | -1.44 | -0.33 | 0.55 | 1.62 |
| 4 | -.83 | -1.69 | -0.79 | 0.13 | 1.37 |
| 5 | .77 | -1.21 | -0.04 | 0.82 | 1.77 |
| 6 | -.80 | -1.69 | -0.72 | 0.11 | 1.23 |

**Suppl. Table 4a.** Standardized single-dimensional and five-dimensional TADS factor models.

| Subscale | Item | 1D Loading | 5D Loading | Thresholds | | | |
| --- | --- | --- | --- | --- | --- | --- | --- |
|  |  |  |  | 1 | 2 | 3 | 4 |
| Physical Neglect | 1 | -.73 | -.80 | -1.90 | -1.03 | -0.25 | 0.56 |
|  | 2 | .44 | .48 | 0.03 | 0.77 | 1.66 | 2.22 |
|  | 4 | .51 | .57 | 0.78 | 1.52 | 2.05 | 2.52 |
|  | 6 | .35 | .39 | 0.24 | 0.69 | 1.36 | 2.22 |
|  | 31 | -.45 | -.50 | -1.61 | -1.21 | -0.77 | -0.03 |
| Emotional Neglect | 5 | -.63 | -.71 | -1.49 | -0.62 | 0.27 | 1.06 |
|  | 8 | -.88 | -.92 | -1.71 | -1.10 | -0.41 | 0.37 |
|  | 13 | -.87 | -.91 | -1.85 | -1.16 | -0.56 | 0.31 |
|  | 21 | -.42 | -.48 | -1.31 | -0.31 | 0.52 | 1.40 |
|  | 40 | -.91 | -.95 | -1.74 | -1.07 | -0.37 | 0.46 |
| Physical Abuse | 9 | .64 | .82 | 0.61 | 0.99 | 1.94 | 2.38 |
|  | 16 | .73 | .89 | 0.89 | 1.19 | 1.97 | 2.62 |
|  | 17 | .47 | .61 | 1.35 | 1.72 | 2.27 | 2.44 |
|  | 20 | .70 | .90 | 0.62 | 1.05 | 1.58 | 2.14 |
|  | 24 | .46 | .61 | 0.38 | 1.04 | 1.89 | 2.98 |
| Emotional Abuse | 10 | .74 | .85 | 0.18 | 0.71 | 1.33 | 1.83 |
|  | 12 | .67 | .77 | 0.14 | 0.57 | 1.25 | 1.94 |
|  | 14 | .48 | .55 | -0.40 | 0.22 | 0.97 | 1.48 |
|  | 26 | .72 | .82 | -0.01 | 0.50 | 1.22 | 1.87 |
|  | 32 | .66 | .74 | -0.26 | 0.20 | 0.86 | 1.66 |
| Sexual Abuse | 22 | .63 | .81 | 1.21 | 1.51 | 1.94 | 2.52 |
|  | 25 | .74 | .90 | 1.41 | 1.68 | 2.18 | 2.32 |
|  | 30 | .85 | .92 | 0.68 | 1.05 | 1.73 | 2.18 |
|  | 33 | .88 | .96 | 0.49 | 0.89 | 1.58 | 2.14 |
|  | 41 | .82 | .92 | 0.84 | 1.19 | 1.74 | 2.38 |

**Suppl. Table 4b.** Factor correlations in the five-dimensional TADS factor model.

| Factor | EMO_NEGL | EMO_ABUS | PHY_NEGL | PHY_ABUS |
| --- | --- | --- | --- | --- |
| Emotional Abuse | 0.80 | – | – | – |
| Physical Neglect | 0.92 | 0.82 | – | – |
| Physical Abuse | 0.52 | 0.68 | 0.75 | – |
| Sexual Abuse | 0.32 | 0.50 | 0.46 | 0.53 |

**Suppl. Table 5.** Results from regression analysis examining the moderation of trauma subtypes on the change in depressive symptoms by resilience.

| Coefficient | Value | SE | *t* | *p* | LLCI | ULCI |
| --- | --- | --- | --- | --- | --- | --- |
| Constant | 9.94 | 6.60 | 1.51 | 0.13 | -3.04 | 22.92 |
| ZTADS | -1.00 | 0.68 | -1.47 | 0.14 | -2.35 | 0.34 |
| ZBRS | 1.88 | 0.64 | 2.93 | 0.004 | 0.62 | 3.14 |
| ZInt1 | 0.81 | 0.55 | 1.47 | 0.14 | -0.28 | 1.91 |
| Age | -0.86 | -2.26 | -2.26 | 0.02 | -1.60 | -0.11 |
| Gender | 1.24 | 1.51 | 0.82 | 0.41 | -1.73 | 4.20 |
|  |  |  |  |  |  |  |
| Constant | 10.46 | 6.55 | 1.60 | 0.11 | -2.42 | 23.34 |
| ZTADSEmNg | -0.80 | 0.66 | -1.21 | 0.23 | -2.08 | 0.49 |
| ZBRS | 1.89 | 0.64 | 2.95 | 0.004 | 0.63 | 3.16 |
| ZInt2 | 0.82 | 0.53 | 1.55 | 0.12 | -0.22 | 1.87 |
| Age | -0.88 | 0.38 | -2.34 | 0.02 | -1.63 | -0.14 |
| Gender | 1.17 | 0.51 | 0.78 | 0.44 | -1.79 | 4.14 |
|  |  |  |  |  |  |  |
| Constant | 9.88 | 6.60 | 1.50 | 0.14 | -3.11 | 22.88 |
| ZTADSEmAb | -0.83 | 0.69 | -1.19 | 0.23 | -0.54 | 2.19 |
| ZBRS | 1.83 | 0.65 | 2.80 | 0.005 | 0.55 | 3.12 |
| Zint3 | -0.62 | 0.56 | -1.11 | 0.27 | -1.72 | 0.48 |
| Age | -0.85 | 0.38 | -2.25 | 0.026 | -1.60 | -0.11 |
| Gender | 1.22 | 1.51 | 0.81 | 0.42 | -1.75 | 4.20 |
|  |  |  |  |  |  |  |
| Constant | 10.10 | 6.55 | 1.54 | 0.12 | -2.78 | 23.00 |
| ZTADSPhyNg | -1.09 | 0.66 | -1.66 | 0.10 | -2.39 | 0.20 |
| ZBRS | 1.86 | 0.64 | 2.91 | 0.004 | 0.60 | 3.12 |
| ZInt4 | 0.92 | 0.54 | 1.69 | 0.09 | -0.15 | 1.96 |
| Age | -0.86 | 0.38 | -2.28 | 0.02 | -0.12 | -1.61 |
| Gender | 1.13 | 1.50 | 0.75 | 0.45 | -1.83 | 4.09 |
|  |  |  |  |  |  |  |
| Constant | 9.82 | 6.56 | 1.50 | 0.14 | -3.09 | 22.73 |
| ZTADSPhyAb | -1.02 | 0.66 | -1.56 | 0.12 | -2.32 | 0.27 |
| ZBRS | 1.91 | 0.63 | 3.01 | 0.003 | 0.66 | 3.16 |
| ZInt5 | 0.82 | 0.58 | 1.41 | 0.16 | -0.32 | 1.96 |
| Age | -0.85 | 0.38 | -2.24 | 0.03 | -1.59 | -0.10 |
| Gender | 1.10 | 1.51 | 0.73 | 0.47 | -1.87 | 4.06 |
|  |  |  |  |  |  |  |
| Constant | 10.65 | 6.71 | 1.59 | 0.11 | -2.56 | 23.86 |
| ZTADSSexAb | -0.32 | 0.68 | -0.47 | 0.64 | -1.66 | 1.03 |
| ZBRS | 2.03 | 0.63 | 3.24 | 0.001 | 0.80 | 3.27 |
| ZInt6 | 0.73 | 0.62 | 1.20 | 0.24 | -0.50 | 1.96 |
| Age | -0.90 | 0.38 | -2.36 | 0.02 | -1.66 | -0.15 |
| Gender | 1.26 | 1.55 | 0.81 | 0.42 | -1.79 | 4.31 |

Abbreviations: BDI = Beck Depression Inventory-IA, BRS = Brief Resilience Scale,
TADS = Trauma and Distress Scale, EmoNg = Emotional neglect, EmoAb = Emotional abuse, PhyNg = Physical neglect, PhyAb = Physical abuse, SexAb = Sexual abuse, ZInt1=ZTADS×ZBRS, ZInt2 = ZTADSEmNg×ZBRS, ZInt3 = ZTADSEmAb×ZBRS, ZInt4 = ZTADSPhyNg×ZBRS, ZInt5 = ZTADSPhyAb×ZBRS, ZInt6 = ZTADSSexAb×ZBRS
