# Supplementary material for: The Effect of Childhood Trauma on the Alleviation of Transdiagnostic Depressive Symptoms and the Mediating Role of Resilience in Outpatient Adolescents
Source: J Child Adolesc Trauma. 2025 Sep 16;18(4):1005–19. doi: 10.1007/s40653-025-00728-8 (PMC12831748; doi:10.1007/s40653-025-00728-8)
Supplement: Supplementary file 2 — Supplementary file2 (DOCX 22 KB) [file 40653_2025_728_MOESM2_ESM.docx]

BDI, BRS, & TADS correlations

2025-04-08

Table of Contents

# Loading R packages and data

## R version 4.4.2 (2024-10-31 ucrt)
## Platform: x86_64-w64-mingw32/x64
## Running under: Windows 10 x64 (build 19045)
##
## Matrix products: default
##
##
## locale:
## [1] LC_COLLATE=Finnish_Finland.utf8 LC_CTYPE=Finnish_Finland.utf8 LC_MONETARY=Finnish_Finland.utf8
## [4] LC_NUMERIC=C LC_TIME=Finnish_Finland.utf8
##
## time zone: Europe/Helsinki
## tzcode source: internal
##
## attached base packages:
## [1] stats graphics grDevices utils datasets methods base
##
## other attached packages:
## [1] stringr_1.5.1 flextable_0.9.7 brunnermunzel_2.0 cocor_1.1-4 psych_2.4.12 dplyr_1.1.4
## [7] haven_2.5.4
##
## loaded via a namespace (and not attached):
## [1] utf8_1.2.4 generics_0.1.3 fontLiberation_0.1.0 xml2_1.3.6
## [5] stringi_1.8.4 lattice_0.22-6 hms_1.1.3 digest_0.6.37
## [9] magrittr_2.0.3 evaluate_1.0.3 grid_4.4.2 fastmap_1.2.0
## [13] zip_2.3.1 pander_0.6.5 fontBitstreamVera_0.1.1 textshaping_0.4.1
## [17] mnormt_2.1.1 cli_3.6.3 crayon_1.5.3 rlang_1.1.4
## [21] fontquiver_0.2.1 withr_3.0.2 yaml_2.3.10 gdtools_0.4.1
## [25] tools_4.4.2 officer_0.6.7 parallel_4.4.2 tzdb_0.4.0
## [29] uuid_1.2-1 forcats_1.0.0 vctrs_0.6.5 R6_2.5.1
## [33] lifecycle_1.0.4 ragg_1.3.3 pkgconfig_2.0.3 pillar_1.10.1
## [37] data.table_1.16.4 glue_1.8.0 Rcpp_1.0.14 systemfonts_1.1.0
## [41] xfun_0.50 tibble_3.2.1 tidyselect_1.2.1 rstudioapi_0.17.1
## [45] knitr_1.49 htmltools_0.5.8.1 nlme_3.1-166 rmarkdown_2.29
## [49] readr_2.1.5 compiler_4.4.2 askpass_1.2.1 openssl_2.3.1

# Comparison of baseline distributions between the follow-up and baseline-only groups

## vars n mean sd median trimmed mad min max range skew kurtosis se
## BDI_t0 1 317 22.74 12.51 23.00 22.61 13.34 0 52 52 0.04 -0.76 0.70
## BRS_t0 2 310 2.78 0.81 2.83 2.75 0.74 1 5 4 0.30 -0.17 0.05
## TADS_t0 3 314 39.64 20.52 38.00 38.62 20.76 2 116 114 0.51 -0.11 1.16
## age_t0 4 319 16.52 1.66 17.00 16.53 1.48 13 19 6 -0.06 -1.20 0.09
## gender_t0 5 319 0.77 0.42 1.00 0.84 0.00 0 1 1 -1.28 -0.35 0.02

## vars n mean sd median trimmed mad min max range skew kurtosis se
## BDI_t0 1 375 22.70 13.01 23.00 22.45 14.83 0 56 56 0.12 -0.74 0.67
## BRS_t0 2 368 2.77 0.79 2.67 2.75 0.74 1 5 4 0.26 0.24 0.04
## TADS_t0 3 368 42.15 24.40 38.50 39.94 22.98 2 126 124 0.81 0.29 1.27
## age_t0 4 380 16.55 1.62 17.00 16.55 1.48 14 20 6 0.04 -1.11 0.08
## gender_t0 5 380 0.70 0.46 1.00 0.75 0.00 0 1 1 -0.87 -1.25 0.02

**Table 6**
*Comparison of baseline distributions between the follow-up and baseline-only groups*

| Variable | Included | | Excluded | | PS | 95% CI | | B-M  statistic | df | p |
| --- | --- | --- | --- | --- | --- | --- | --- | --- | --- | --- |
|  | Mean/% | SD | Mean/% | SD |  | Lower | Upper |  |  |  |
| BDI | 22.74 | 12.51 | 22.70 | 13.01 | .497 | .454 | .540 | -0.141 | 680 | .888 |
| BRS | 2.78 | 0.81 | 2.77 | 0.79 | .502 | .458 | .546 | 0.083 | 635 | .934 |
| TADS | 39.64 | 20.52 | 42.15 | 24.40 | .515 | .471 | .558 | 0.669 | 679 | .504 |
| Age | 16.52 | 1.66 | 16.55 | 1.62 | .504 | .461 | .546 | 0.164 | 664 | .870 |
| Female | 77.1% | - | 70.0% | - | .464 | .432 | .497 | -2.137 | 692 | .033 |
| SD = Standard Deviation; PS = Probability of Superiority: P(X<Y) + 0.5 * P(X=Y);  CI = Confidence Interval; B-M = Brunner-Munzel test; df = Degrees of freedom | | | | | | | | | | |

# Comparison of baseline correlations between the follow-up and baseline-only groups

**Table 7**
*Comparison of baseline correlations between the follow-up and baseline-only groups*

| Variables | | Correlations | | Correlation  difference | 95% CI | | Fisher's test | |
| --- | --- | --- | --- | --- | --- | --- | --- | --- |
|  |  | Included | Excluded |  | Lower | Upper | z | p |
| BDI | BRS | -.529 | -.550 | 0.021 | -0.086 | 0.130 | 0.390 | .697 |
| BDI | TADS | .493 | .541 | -0.048 | -0.160 | 0.062 | -0.851 | .395 |
| BRS | TADS | -.316 | -.339 | 0.023 | -0.112 | 0.159 | 0.335 | .738 |
| CI = Confidence Interval | | | | | | | | |
